# Supplementary material for: An EpCAM/Trop2 mechanostat differentially regulates collective behaviour of human carcinoma cells
Source: EMBO J. 2024 Nov 21;44(1):75–106. doi: 10.1038/s44318-024-00309-9 (PMC11696905; doi:10.1038/s44318-024-00309-9)
Supplement: Supplementary file 1 — Appendix [file 44318_2024_309_MOESM1_ESM.pdf]

Appendix

An EpCAM/Trop2 mechanostat differentially regulates collective behaviour of human carcinoma cells

Aslemarz A, Fagotto-Kaufmann M, Ruppel A, Fagotto-Kaufmann C, Balland M, Lasko P, and Fagotto F

Table of content

Appendix Fig. S1
Comparative expression of EpCAM and Trop2 in various cancer lines. 2

Appendix Fig. S2
Estimate of relative endogenous EpCAM and Trop2 levels in MCF7 cells. 3

Appendix Fig. S3
Total EpCAM and Trop2 levels in MCF7 cells in control, EpCAM KD and Trop2 KD. 4

Appendix Fig. S4
Contact angles of single cells on collagen gel. 5

Appendix Fig. S5
Details of pMLC and phalloidin staining of spheroids. 6

Appendix Fig. S6
F-actin quantification for small MCF7 cell groups. 7

Appendix Fig. S7
Spheroid spreading on collagen-coated 5kPa polyacrylamide gel 8

Appendix Fig. S8
Analysis of EpCAM and Trop2 cell surface distribution in MCF7 cells 9

Appendix Section 1: CompuCell3D Simulation 11

Aslemarz et al. Appendix Figure S1

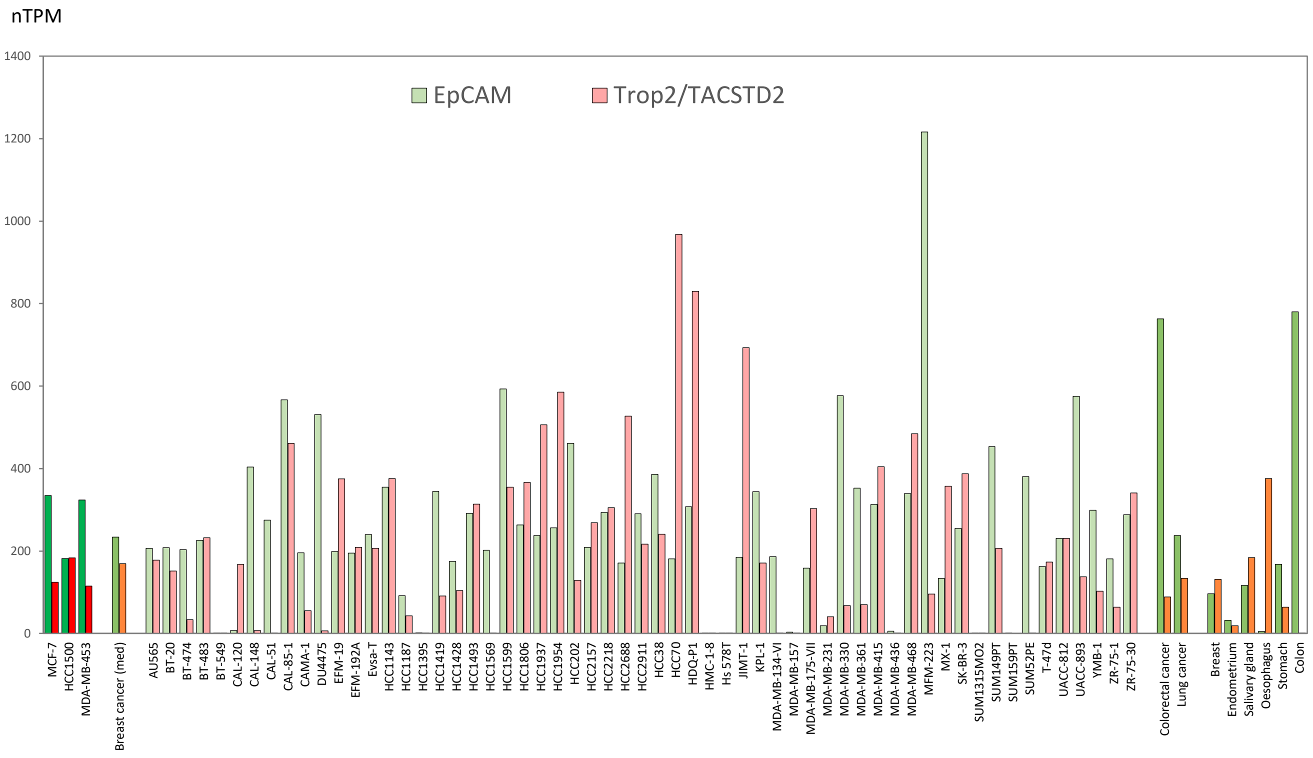

Appendix Figure S1. Comparative expression of EpCAM and Trop2 in various cancer lines.

The list includes three cell lines used in this study (MCF7, HCC1500 and MDA-MB-453) with median of 62 breast cancer cell lines, individual values for each lineRNA-seq data were extracted from the Human Protein Atlas database (https://www.proteinatlas.org). Additional data on the left include the medians for colon and lung cancer cell lines (respectively 63 and 232 lines), as well as values for normal tissues (data from HPA RNA-seq, also in Human Protein Atlas). nTPM: Normalized transcripts per million.

Aslemarz et al. Appendix Figure S2

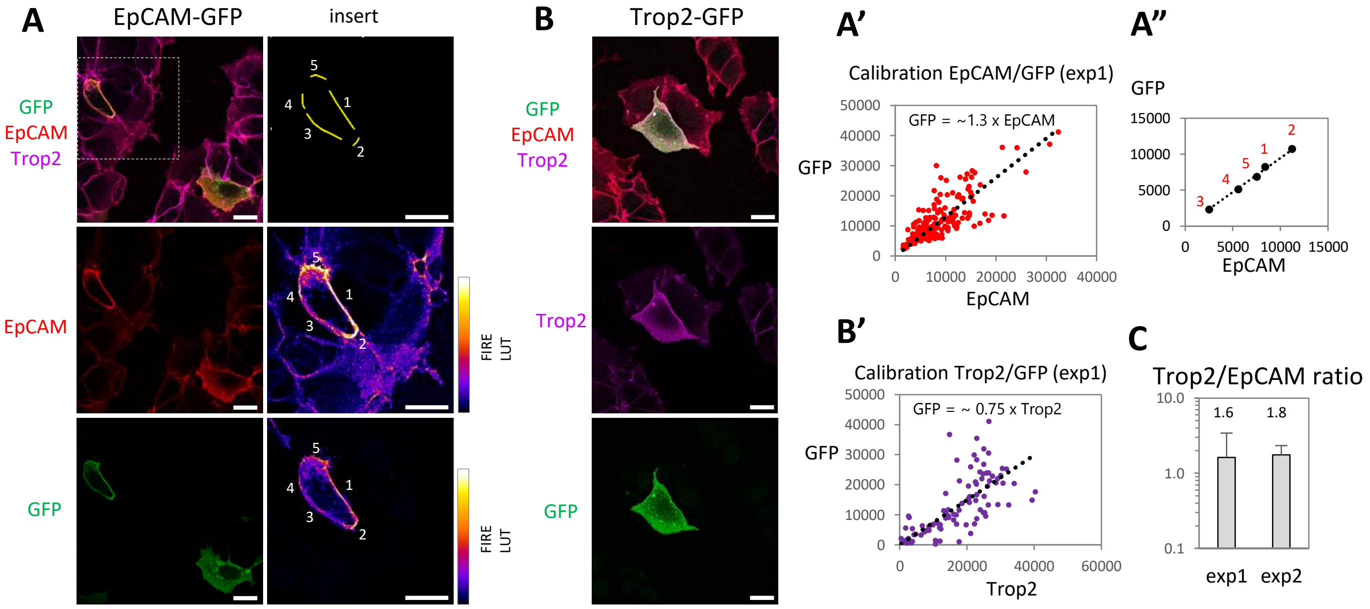

Appendix Figure S2. Estimate of relative endogenous EpCAM and Trop2 levels in MCF7 cells.

The strategy was to use the GFP signal of overexpressed EpCAM-GFP and Trop2-GFP to calibrate the respective signals of the anti-EpCAM and anti-GFP antibodies. Using these calibrations, we could calculate an estimated ratio of endogenous EpCAM and Trop2 in double stained cells. MCF7 cells were transfected with DOX-inducible EpCAM-GFP and Trop2-GFP constructs, treated overnight with 1μM DOX, fixed and immunolabelled, without permeabilization, for EpCAM and Trop2, then postfixed and permeabilized, and immunolabelled for GFP (positioned at the cytoplasmic C-terminus).

(**A**,**B**) Examples of confocal images, showing individual transfected cells among wild type MCF7 cells. (**Insert**) Example of measurement on one EpCAM-GFP positive cell: 6 membrane segments of various intensities, numbered 1-6, were used to quantify GFP and EpCAM signal intensities plotted in A”.

(**A’**,**B’**) Compilation of these values for experiment 1 (6 images for each condition), which were used to determine the approximative slopes. (**C**) These slopes allowed to directly compare endogenous EpCAM and Trop2 levels based on the signal intensities in non-transfected cells. Results from 2 experiments, 12 fields for each. Average values are indicated on a log scale. Bars: SD. This is a rough estimate, as levels strongly varied between cells, even though the GFP to EpCAM/Trop2 signal ratio within individual cells often resulted quite linear (A”). Scale bars: 20μm.

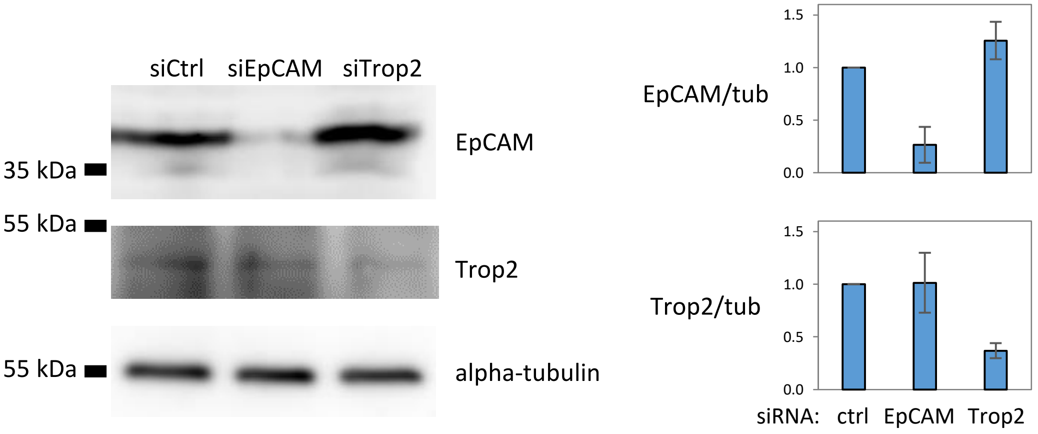

Appendix Figure S3.

Total EpCAM and Trop2 levels in MCF7 cells in control, EpCAM KD and Trop2 KD.

Lysates of MCF7 confluent monolayers were analysed by Western Blot. Alpha-tubulin was used as loading control, and the intensity ratios were normalized to siCtrl. Average of three independent experiments. Error bars, SD.

Aslemarz et al. Appendix Figure S4

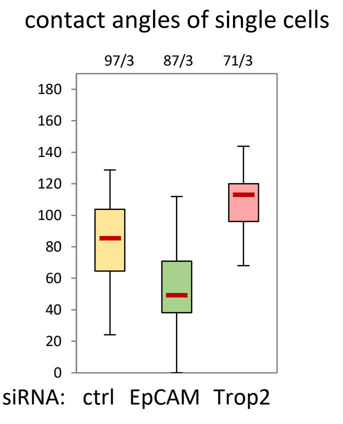

Appendix Figure S4. Contact angles of single cells on collagen gel.

Contact angles were measured from z projections of phalloidin stained cells. These are only estimates, as the highly irregular shapes and presence of numerous protrusions precluded accurate measurements. The box plots show the interquartile range (box limits), median (center line), and min and max values without outliers (whiskers). Numbers of cells/biological replicates indicated above graphs.

Aslemarz et al. Appendix Figure S5

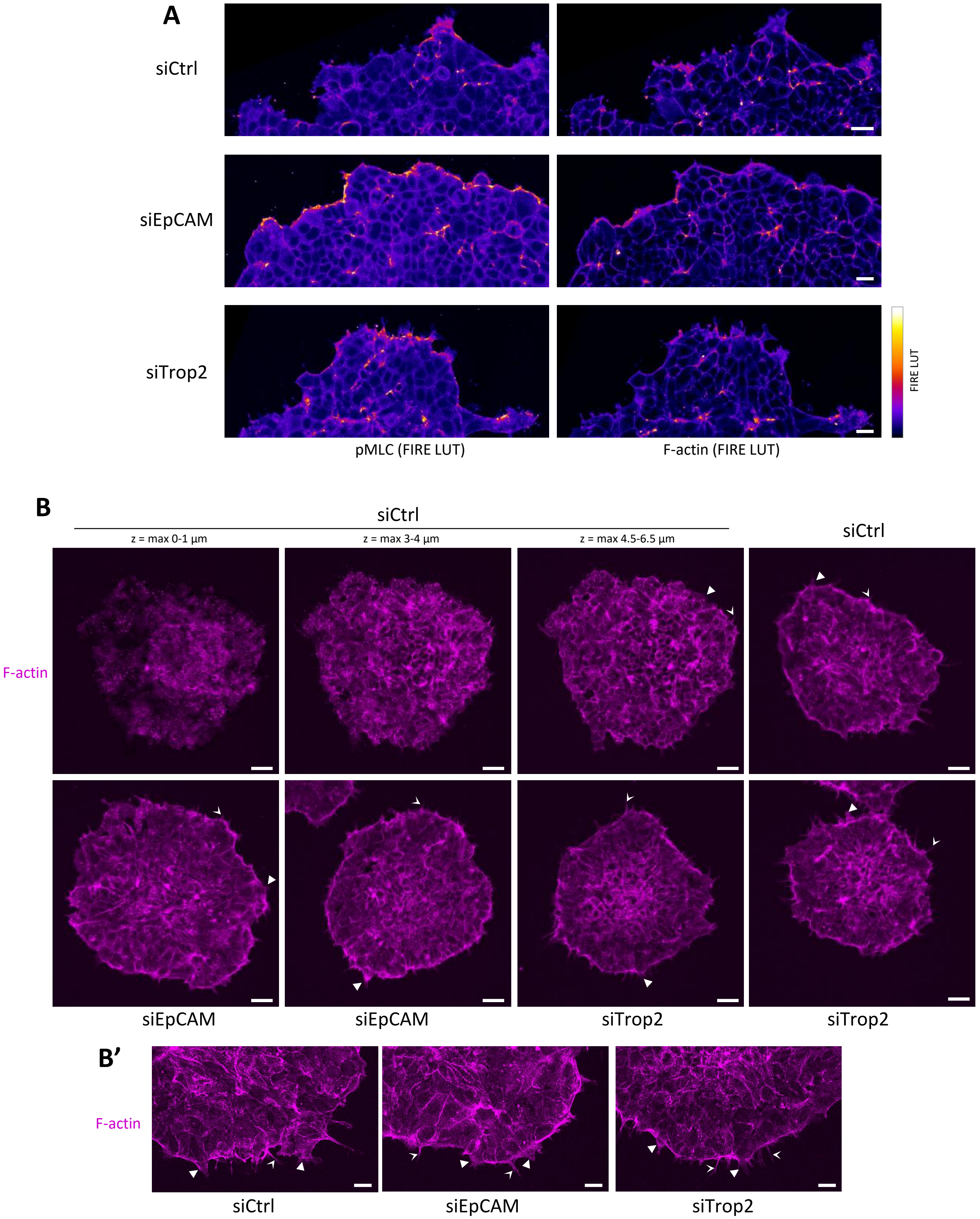

Appendix Figure S5. Details of pMLC and phalloidin staining of spheroids.

(**A**) **Enlarged views of p**eripheral regions of spheroids stained pMLC and phalloidin. Selected planes correspond to the broadest area of each spheroid showed, just above the collagen surface. (**B**) Confocal images of small spheroids stained with phalloidin. Top three left images show three slices of the bottom of a spheroid, to illustrate the convex and complex structure of the ventral interface in contact with the collagen matrix. Strong stress fibres are lacking, which is expected with a soft substrate. The other images illustrate the F-actin patterns just above the collagen surface, revealing the dense peripheral cortex. A variety of protrusions were abundant at the edge of all spheroids. Filled arrowheads: large protrusion. Concave arrowheads: thin protrusions. (B') Enlarged views. Scale bars: A, 20µm; B, 50µm; B', 20µm.

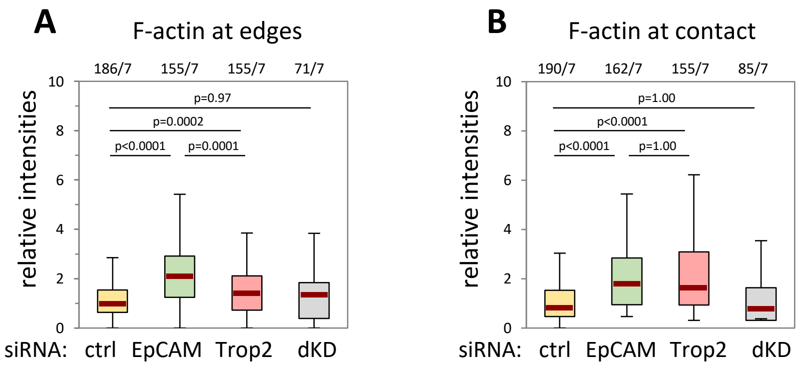

Appendix Figure S6. F-actin quantification for small MCF7 cell groups.

Peak phalloidin signal intensity at free edges and cell-cell contacts, normalized to the average signal at edges. The box plots show the interquartile range (box limits), median (center line), and min and max values without outliers (whiskers). Numbers of cell groups/biological replicates indicated above graphs. Statistics: one-way non-parametric ANOVA (Kruskal-Wallis Test) followed by post-hoc Dunn tests.

Aslemarz et al. Appendix Figure S7

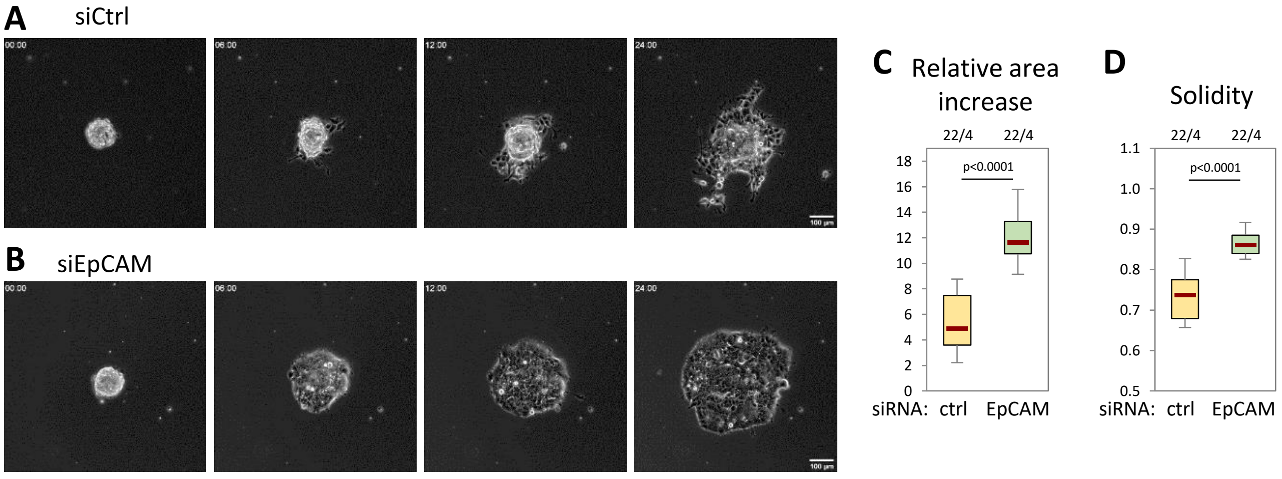

Appendix Figure S7. Spheroid spreading on collagen-coated 5kPa polyacrylamide gel

A,B. Images of siCtrl and siEpCAM spheroids at four time points as in Figure 1. EpCAM KD spheroids spread much more than controls. Control spheroids are very irregular, with cells at the edge bulging out and even detaching. EpCAM KD spheroids remain much more coherent. Scale bars: 150µm.

C,D. Quantification of area increase and solidity, as in main Fig.1. The box plots show the interquartile range (box limits), median (center line), and min and max values without outliers (whiskers). 22 spheroids per conditions from four independent experiments. Statistical analysis: paired, two-tailed Student's t-test.

Aslemarz et al. Appendix Figure S8

Analysis of EpCAM and Trop2 cell surface distribution in MCF7 cells

A

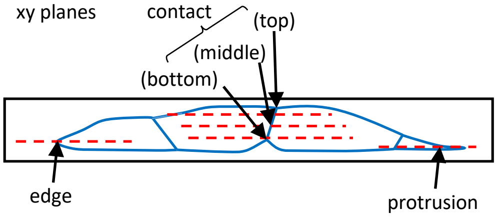

A'

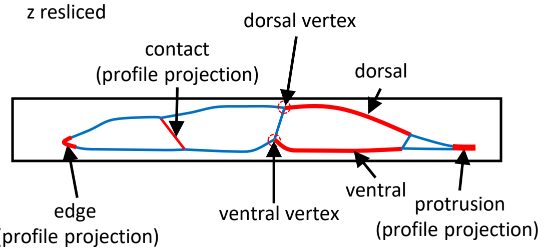

B Distribution of EpCAM and Trop2 in cell surface regions of MCF7 cells (Relative levels normalized to ave contacts)

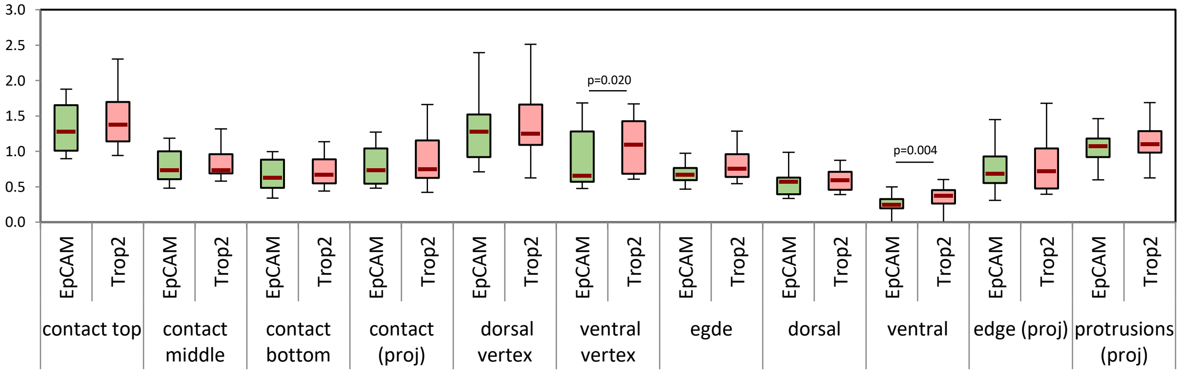

C

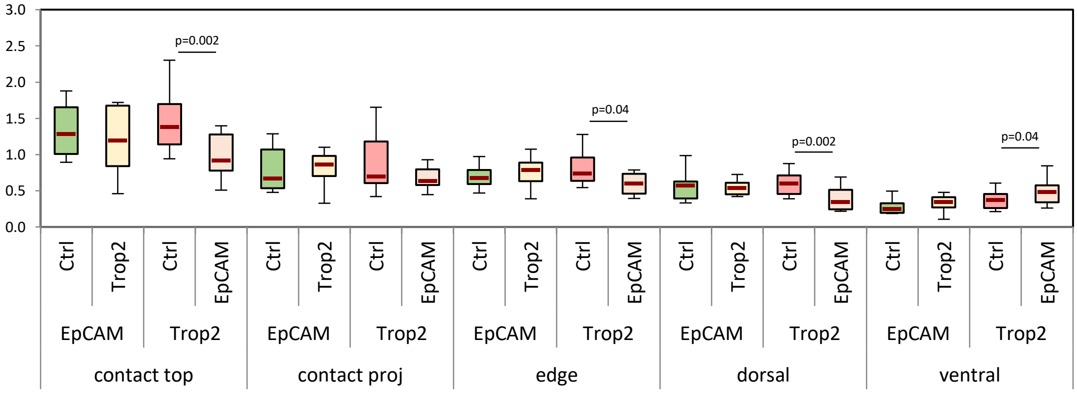

D EpCAM/Trop2 distribution in large group of MCF7 cells (non-permeabilized)

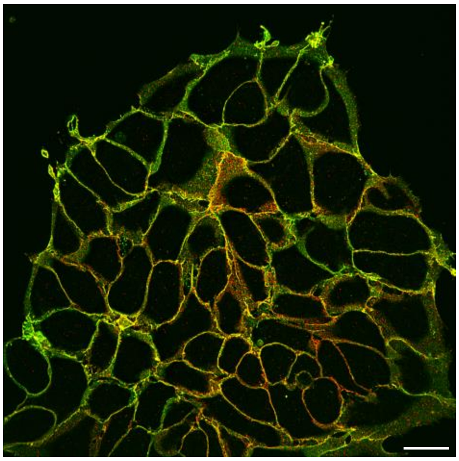

EpCAM Trop2

E EpCAM/Trop2 distribution (post-fixation permeabilization)

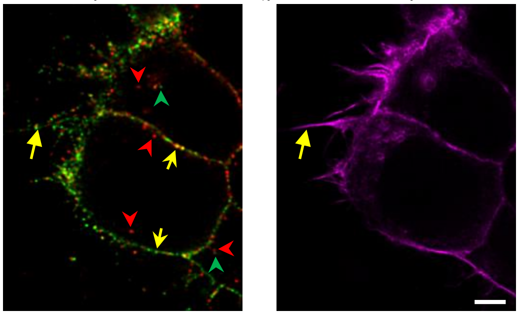

EpCAM Trop2 F-actin

Appendix Figure S8. Detailed analysis of EpCAM and Trop2 distribution in MCF7 cells.

A. Schematic representation of a group of cells, viewed in profile, showing the typical position of horizontal (xy) planes used to measure line fluorescence intensities along edge and contact cell membrane. **A'.** Same scheme, showing regions measured in profile projections, obtained by reslicing the image stacks. Intensities were also measured from lines, except for vertices, for which small circles were used. A total of 11 regions were measured. Values of each experiment were normalized to the average value from of the three regions of cell-cell contacts in xy sections, taken near the top, middle and bottom of each contact (bracket in A).

B. Quantification of EpCAM and Trop2 levels in control MCF7 cells. Note that several of the categories corresponded to the overlapping regions, measured either in the original horizontal planes or in the corresponding profile projections. The two types of measurements show high consistency, e.g. cell edges and cell protrusions (also compare average contact in xy planes versus whole contacts in profile projections). EpCAM and Trop2 showed quasi identical distributions. The only statistically significant difference was found along the ventral membrane. The box plots show the interquartile range (box limits), median (center line), and min and max values without outliers (whiskers). 17 groups of cells from three independent experiments. Statistical analysis: Student's t-test. Only statistically significant pairs are indicated.

C. Effect of EpCAM depletions on Trop2 levels and, reciprocally, of Trop2 depletion on EpCAM levels, for selected regions of the cell membrane. Neither EpCAM levels nor its distribution are significantly changed by Trop2 depletions. Upon EpCAM KD, Trop2 distribution is modified at four localization: Trop2 levels drop at lateral edges and dorsal side, including the dorsal region of cell-cell contacts (contact top), but not average levels along the whole cell-cell contact (profile). Trop2 levels are prominently increased at the ventral side. The box plots show the interquartile range (box limits), median (center line), and min and max values without outliers (whiskers). 15,13 and 12 groups of cells for siCtrl, siEpCAM and siTrop2, from three independent experiments Student's t-test between control and corresponding KD condition. Only statistically significant pairs are indicated. Student's t-test.

D. Example of large cell group showing a typical enrichment of Trop2 along internal contacts compared to EpCAM. Maximal projection of 7 planes, total thickness 1.5µm. Scale bar: 20µm.

E. Example of immunofluorescence of EpCAM and Trop2 in cells permeabilized with 0.2% Triton X100 after fixation. Both signals along the plasma membrane (yellow arrows) are more punctate compared to those obtained without permeabilization (Fig 8 and panel F). Yellow filled arrows: Punctate signal at protrusions. Trop2 signal at the plasma membrane was much lower than using surface labelling without permeabilization. Few intracellular spots positive for EpCAM or Trop2 are detected (green and red arrowheads). Scale bar: 5µm.

Appendix Section 1: CompuCell3D Simulation

All Simulations were performed with CompuCell3D, a software package that simulates cell behavior in a 3D environment using a Cellular Potts Model. For a detailed description of the model, please see the corresponding publication (Swat et al, 2012).
In short, the model consists of a regular, 3D lattice where each pixel belongs to a specific, user-defined generic cell. These cells can correspond to actual cells, the substrate, the medium or other components and the user can assign attributes to these cells and their interactions with other cells. Next, the users specifies an initial configuration, which then evolves over time according to the user-defined interaction rules.
The evolution of the initial configuration proceeds by minimizing the overall energy in the system. More concretely, in each simulation step, some pixels change their state randomly, where the number of pixels that change per step is defined with the temperature parameter, and the overall energy of the new configuration is calculated. If the energy of the new configuration is smaller than the energy of the previous configuration, the new configuration is accepted. If the energy is higher, the new configuration is accepted with a probability defined by a Hamiltonian, a function that is the sum of energies resulting from various parameters, in particular energies at contact interfaces.
In our case, there were three different types of cells: the substrate, the medium and the cells. The interaction between the different cell types were defined by their corresponding binding energies. This yielded 6 different parameters in addition to the temperature parameter and the total number of simulation steps:

-Medium-medium energy
-Medium-cell energy
-Medium-substrate energy
-Cell-cell energy
-Cell-substrate energy
-Substrate-substrate energy

Since both the medium and the substrate are immobile, i.e. they never switch state unless replaced by a cell, their interactions with themselves and with each other don't impact the result of the simulation. In other words, the medium-medium and the substrate-substrate energy parameter are irrelevant. From the four remaining parameters, only three are independent. Since the system only tries to minimize the overall energy by comparing different states, the total energy of the system is arbitrary. Therefore, we can set one of these parameters to 0 without losing any degrees of freedom. We chose to set the medium-substrate energy to 0, which leaves us with three remaining free parameters: The cell-cell, the cell-substrate and the cell-medium energy.
Choosing these three parameters has the advantage that they all correspond to concrete physical properties of the cells. The cell-medium energy corresponds to the cell cortical tension, the cell-cell energy is inversely related to cell-cell adhesiveness and the cell-substrate energy to cell-substrate adhesiveness. This direct correspondence allowed us to identify the parameters for the different conditions one by one. First, we set the cell-medium energy for siEpCAM arbitrarily to 4. We approximated the values for other conditions, 2 for siCtrl and 3.5 for siTrop2, based on data including contractile energies from TFM, pMLC and vinculin IF.
Values for cell-matrix energy relative to cell-medium energy were estimated using the morphology of single cells on matrix, modelled as disk segments (Figure 5E), the secant corresponding to the diameter, based on the measurements of Figure 1I. We verified that these simplified "average" shapes had contact angles (90, 75 and 110° for siCtrl, siEpCAM and siTrop2, respectively) fitting within the range measured experimentally (Appendix Figure S5), although the latter measurements were much less accurate. For cell-cell contact energy, we modelled the angle at the vertex of free doublets, which is directly related to adhesiveness (Fig. 4H). We matched CPM simulations to approach these values, by varying the cell-substrate energy. To deal with the stochastic and pixelated nature of the simulation, we calculated an average image over 50 simulation time points (Fig. 5E,E'). Note that these were approximations, since angles were difficult to measure on these pixelated, averaged images.
The final parameters were:

	siCtrl	siEpCAM	siTrop2
Cell-medium energy	2	4	3.5
Cell-substrate energy	0	-2	1
Cell-cell energy	3	5	6

These values were in agreement with the ensemble of quantitative and semi-quantitative of this study.

We used these parameters to simulate spreading spheroids of approximately 100 cells and measured the spreading surface over time, normalized by the initial area, as well as the solidity of the spreading surface, which is a measure of the roughness of the contour (see Annex figure 2).
Altogether, these results show that the spheroid phenotype is consistent with the single cell and free doublet phenotype and that higher cell contractility can lead to very different outcomes, depending on how cell-cell and cell-substrate adhesion is altered.

References

Swat MH, Thomas GL, Belmonte JM, Shirinifard A, Hmeljak D & Glazier JA (2012) Multi-scale modeling of tissues using CompuCell3D. Methods Cell Biol 110: 325–366
