# Supplementary figures and images for: An EpCAM/Trop2 mechanostat differentially regulates collective behaviour of human carcinoma cells
Source: EMBO J. 2024 Nov 21;44(1):75–106. doi: 10.1038/s44318-024-00309-9 (PMC11696905; doi:10.1038/s44318-024-00309-9)

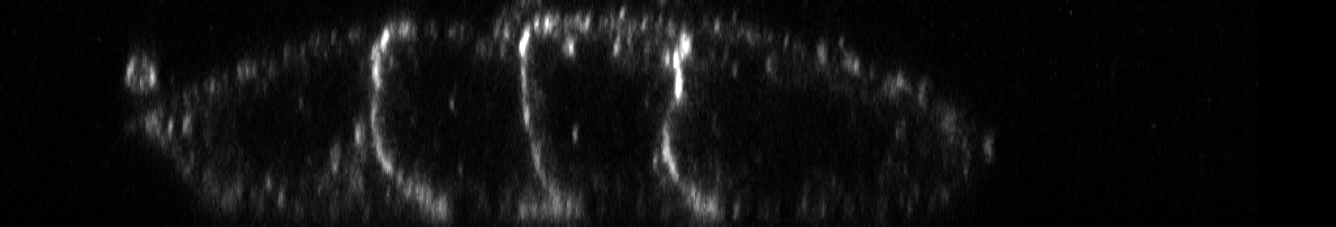

Supplement: Supplementary file 9 — Source data Fig. 2G1 [file 44318_2024_309_MOESM9_ESM.zip › 2G1/confocal 2G''' siCtrl_orthogonal 1.tif]

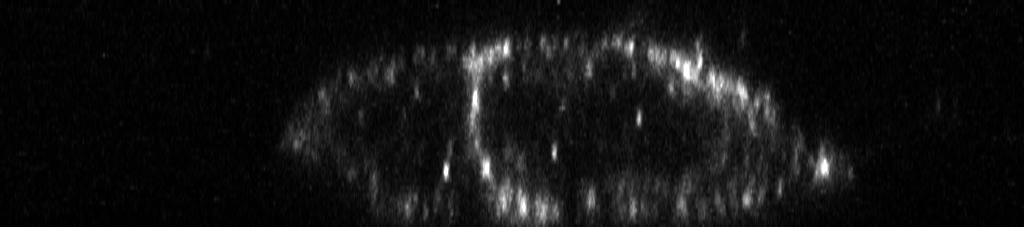

Supplement: Supplementary file 9 — Source data Fig. 2G1 [file 44318_2024_309_MOESM9_ESM.zip › 2G1/confocal 2G''' siCtrl_orthogonal 2.tif]
