# Supplementary material for: An EpCAM/Trop2 mechanostat differentially regulates collective behaviour of human carcinoma cells
Source: EMBO J. 2024 Nov 21;44(1):75–106. doi: 10.1038/s44318-024-00309-9 (PMC11696905; doi:10.1038/s44318-024-00309-9)
Supplement: Supplementary file 10 — Expanded View Figures [file 44318_2024_309_MOESM10_ESM.pdf]

Expanded View Figures

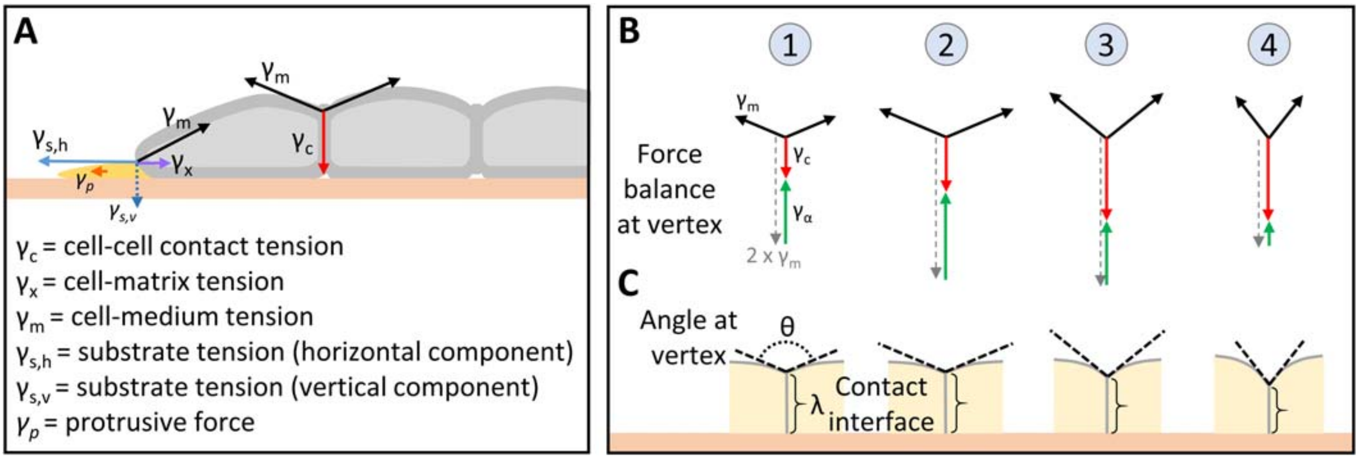

Figure EV1. Summary of the biophysical description of cell spreading and adhesion based on the balance of tensions at interfaces.

(**A**) The capacity of cells to spread on a substrate, be it matrix or other cells, is strongly dependent on the contractility of the actomyosin cortex, which can be viewed as analogous to the role of surface tension in the physical process of a liquid wetting a surface (Brodland, 2002; Douezan et al, 2011; Amack and Manning, 2012; Winklbauer, 2015). The system can similarly be described as the balance of the tensions exerted at various interfaces, namely tension at the matrix (γ_x), at cell contact (γ_c), and along the free edges exposed to the medium (γ_m). γ_x and γ_c combine various tensions exerted at the corresponding interface. In the case of cell-cell adhesion, for instance, γ_c is the sum of the cortical tensions on each side of the contact minus the so-called "adhesive tension" (see below). For adhesion to matrix, γ_x and γ_m are balanced by substrate tension γ_s. Note that it is customary to consider the horizontal components $\gamma_{s,h}$, which is typically the one measured by techniques such as traction force microscopy. Spreading of cell aggregates is controlled by the combination of cell-matrix and cell-cell adhesion, thus by all three tensions (Ryan et al, 2001; Douezan et al, 2011). Actin polymerization within the advancing protrusion also generates a force γ_p, which may contribute to the force balance, depending on the degree of the mechanical coupling with the rest of the cytoskeleton. (**B**) Cells and tissues constitute active materials, and their behavior is clearly more complex than the wetting/adhesion of a classical liquid. Taking the case of cell-cell adhesion, expansion of an adhesive contact λ requires that contact tension γ_c is lowered compared to the basal cortical tension γ_m that would otherwise act on each side of the interface (2 x γ_m). This is achieved partly by the adhesion tension resulting from the binding of adhesion molecules to their ligands (cadherin-cadherin and integrins-matrix), but mainly through an active downregulation of local actomyosin contractility along the contact interface. This latter process relies on the ability of adhesion molecules to recruit regulators of cytoskeleton remodeling. To highlight this downregulation, we introduce here a single tension vector γ_α that includes the ensemble of contributions that decrease γ_c (thus $2\gamma_m = \gamma_c - \gamma_\alpha$). Cadherin coupling to the actomyosin network (Leckband and de Rooij, 2014; Charras and Yap, 2018), and stress fibers anchoring focal adhesions for matrix adhesion, are important additional inputs that impact tensions at the various interfaces. (**C**) Cell geometry reflects the force balance: As shown in panel (1), a low γ_c/γ_m ratio (high γ_α) leads to a large contact interface λ, and a wide-angle θ. The latter is a direct geometric readout of "adhesiveness" (David et al, 2014). A corollary is that a smooth surface directly reflects the high cohesivity of a tissue (Amack and Manning, 2012; Winklbauer, 2015). (2–4) Contacts adapt to stress through a process of reinforcement, involving the recruitment of cortical cytoskeleton and of adhesion molecules, and increased linkage of adhesion molecules to the cytoskeleton (Engl et al, 2014; Charras and Yap, 2018). As shown in panel (2), cells can then maintain the same degree of adhesiveness (same angle θ) despite bearing higher tensions. Importantly, this also requires more repression of contractility along the cell-cell interface (increased γ_α), another expected effect of enhanced cadherin recruitment. Panel (3) shows the situation where higher cortical tension is not compensated by increased γ_α, resulting in a higher γ_c/γ_m ratio and lower adhesiveness (smaller angle θ). In the last example (4), γ_m remains unchanged compared to (1), but γ_α is decreased, resulting in relatively high γ_c, and thus low adhesiveness (low angle θ and shorter contact interface λ. The same principles are applicable to adhesion and spreading to the extracellular matrix, which depends on the balance between γ_x and γ_m. Here, in addition to cortical contractility, the contribution of stress fibers also impacts the balance between γ_x and γ_m.

 © The Author(s)

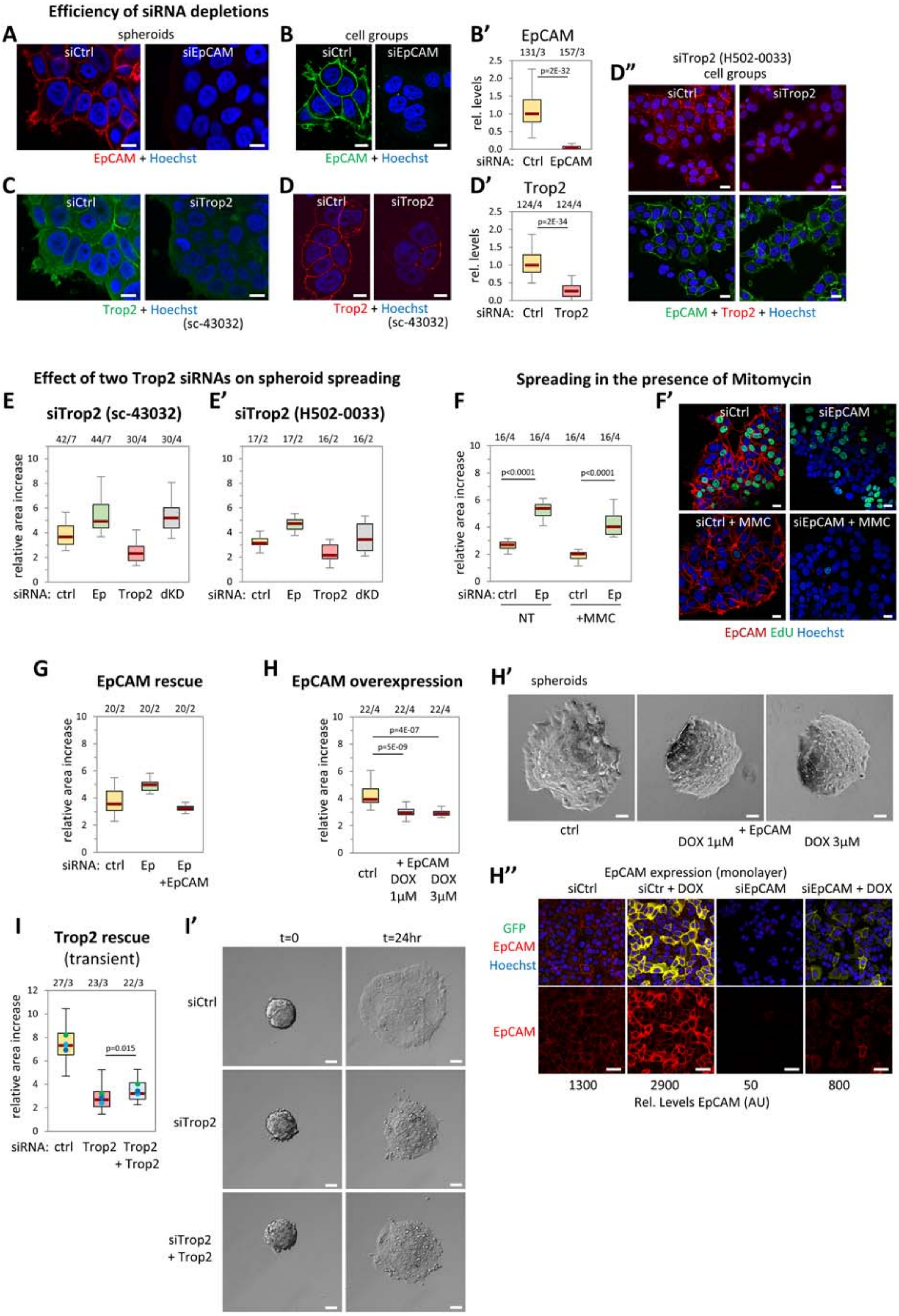
Efficiency of siRNA depletions
A spheroids
siCtrl siEpCAM
EpCAM + Hoechst
B cell groups
siCtrl siEpCAM
EpCAM + Hoechst
B' EpCAM
131/3 157/3
p=2E-32
rel. levels
siRNA: Ctrl EpCAM
C siCtrl siTrop2
Trop2 + Hoechst (sc-43032)
D siCtrl siTrop2
Trop2 + Hoechst (sc-43032)
D' Trop2
124/4 124/4
p=2E-34
rel. levels
siRNA: Ctrl Trop2
D'' siTrop2 (H502-0033) cell groups
siCtrl siTrop2
EpCAM + Trop2 + Hoechst
Effect of two Trop2 siRNAs on spheroid spreading
E siTrop2 (sc-43032)
42/7 44/7 30/4 30/4
relative area increase
siRNA: ctrl Ep Trop2 dKD
E' siTrop2 (H502-0033)
17/2 17/2 16/2 16/2
siRNA: ctrl Ep Trop2 dKD
Spreading in the presence of Mitomycin
F
16/4 16/4 16/4 16/4
p<0.0001 p<0.0001
relative area increase
siRNA: ctrl Ep ctrl Ep
NT +MMC
F' siCtrl siEpCAM
siCtrl + MMC siEpCAM + MMC
EpCAM EdU Hoechst
G EpCAM rescue
20/2 20/2 20/2
relative area increase
siRNA: ctrl Ep Ep +EpCAM
H EpCAM overexpression
22/4 22/4 22/4
p=4E-07
p=5E-09
relative area increase
ctrl + EpCAM DOX 1µM DOX 3µM
H' spheroids
ctrl + EpCAM DOX 1µM DOX 3µM
H'' EpCAM expression (monolayer)
siCtrl siCtr + DOX siEpCAM siEpCAM + DOX
GFP EpCAM Hoechst
EpCAM
1300 2900 50 800
Rel. Levels EpCAM (AU)
I Trop2 rescue (transient)
27/3 23/3 22/3
p=0.015
relative area increase
siRNA: ctrl Trop2 Trop2 + Trop2
I' t=0 t=24hr
siCtrl
siTrop2
siTrop2 + Trop2

◀ **Figure EV2. EpCAM and Trop2 depletions, rescues, and other controls.**

(**A–D**) EpCAM and Trop2 depletions. (**A, C**) Representative confocal microscopy images of the edge of spheroids formed with cell transfected for 96 h with Ctrl, EpCAM, and Trop2 siRNA (sc-43032), immunolabelled for EpCAM and Trop2. Nuclei were stained with Hoechst (blue). The specific signal along cell membranes is undetectable in the respective siRNA condition. (**B**, **D**) Confocal microscopy images of groups of cells transfected for 96 h with Ctrl, EpCAM, and Trop2 siRNA (sc-43032), immunolabelled for EpCAM and Trop2. Scale bars: 10 μm. (**B′**, **D′**) Quantification of EpCAM and Trop2 signal intensity at cell-cell contacts. Levels were normalized to the median value for siCtrl cells. The box plots show the interquartile range (box limits), median (center line), and min and max values without outliers (whiskers). Numbers of individual contacts/biological replicates indicated above graphs. Statistical analysis, two-tailed Student's *t*-test. (**D″**) Same staining as in (**D**), for cells transfected with siCtrl and siTrop2 (H502-0033). Scale bars: 20 μm. (**E**) Comparison of the effect of siTrop2 sc-43032 and H502-0033 on spheroid spreading. Numbers of spheroids/biological replicates indicated above graphs (in Source Data, (**E**) = exp1–3,6–9, (**E′**) = exp4,5). Statistical analysis: one-way ANOVA followed by Tukey-HSD post hoc test. (**E**″). Immunofluorescence for EpCAM and Trop2 of groups of control and H502-0033-transfected cells. (**F**) Increased spheroid migration upon EpCAM KD is independent of cell proliferation. Spheroids were treated with 2.5 μM mitomycin C (MMC) during the entire migration assay. Numbers of spheroids/biological replicates indicated above graphs. Statistical analysis: one-way ANOVA followed by Tukey-HSD post hoc test. (**F′**) Validation of mitomycin MMC efficiency by imaging EdU incorporation. At the end of the migration assay, the spheroids were incubated for 1 h with thymidine analog EdU, which efficiently incorporates into newly synthesized DNA. EdU was detected in green (see Material and Methods), while EpCAM was detected by immunofluorescence (red), and nuclei were stained with Hoechst (blue). The four panels show representative confocal microscopy images of non-treated, and MMC-treated spheroids of siCtrl and siEpCAM conditions. Maximal projections of 3 z planes, 0.5 m apart. Scale bars: 20 μm. (**G**) Rescue of EpCAM KD spheroid phenotype. Rescue of spheroid spreading phenotype (24 h) was performed using a mixed population of MCF7 cells stably transfected with a doxycycline (DOX)-inducible EpCAM-GFP variant that included two conservative point mutations within the siRNA target sequence. Ctrl, transfected with siCtrl, no DOX; siEpCAM, no DOX; siEpCAM + 1 μM DOX. Under these conditions, control and siEpCAM spheroids spread to a similar extend as, respectively, regular control and EpCAM KD spheroids (Fig. 1A). DOX treatment fully rescued spreading to control levels. Results from 20 spheroids per conditions, two independent experiments. (**H**) EpCAM overexpression inhibits spheroid spreading. Spheroids of cells expressing doxycycline-inducible EpCAM-GFP were let spreading for 24 h on collagen gel. Conditions included non-treated control spheroids, and spheroids treated with 1 μM and 3 μM DOX. Results of 22 spheroids, from four independent experiments. Statistical analysis: one-way ANOVA followed by Tukey-HSD post hoc test. (**H′**) Representative examples for the three conditions, 24 h spreading. Scale bars, 50 μm. (**H″**) Representative EpCAM immunofluorescence images of control, DOX-induced, EpCAM-depleted (siEpCAM) and rescued (siEpCAM + DOX) cells. Cells were double labeled for EpCAM, GFP (to detect exogenous, DOX-induced EpCAM). Total relative EpCAM levels in each image are indicated on the right. Scale bars, 50 μm. (**I**) Transient expression of Trop2-GFP partially rescues the Trop2 KD spheroid phenotype. Spheroids of MCF7 cells transfected consecutively with siTrop2 and a DOX-inducible Trop2-GFP construct, were laid on collagen gel and left to spread in the absence or in the presence of 0.5–3 μM DOX. Positive controls were transfected with siCtrl. Note that spheroids were much smaller than in the other experiments, being formed with 100 rather than 400 cells, and expanded more extensively (~7 folds versus ~4 folds). The reason for this protocol modification was that larger spheroids were more severely damaged by cell death caused by the transient transfection of the Trop2-GFP plasmid, independently of DOX induction. Results from 22–27 spheroids from three independent experiments. Statistical comparison, Student's *t*-test, two-tailed. (**I′**) Examples of control, Trop2 KD (siTrop2 + Trop2-GFP without DOX) and rescue ((siTrop2 + Trop2-GFP with 3 μM DOX). Scale bars, 50 μm.

 © The Author(s)

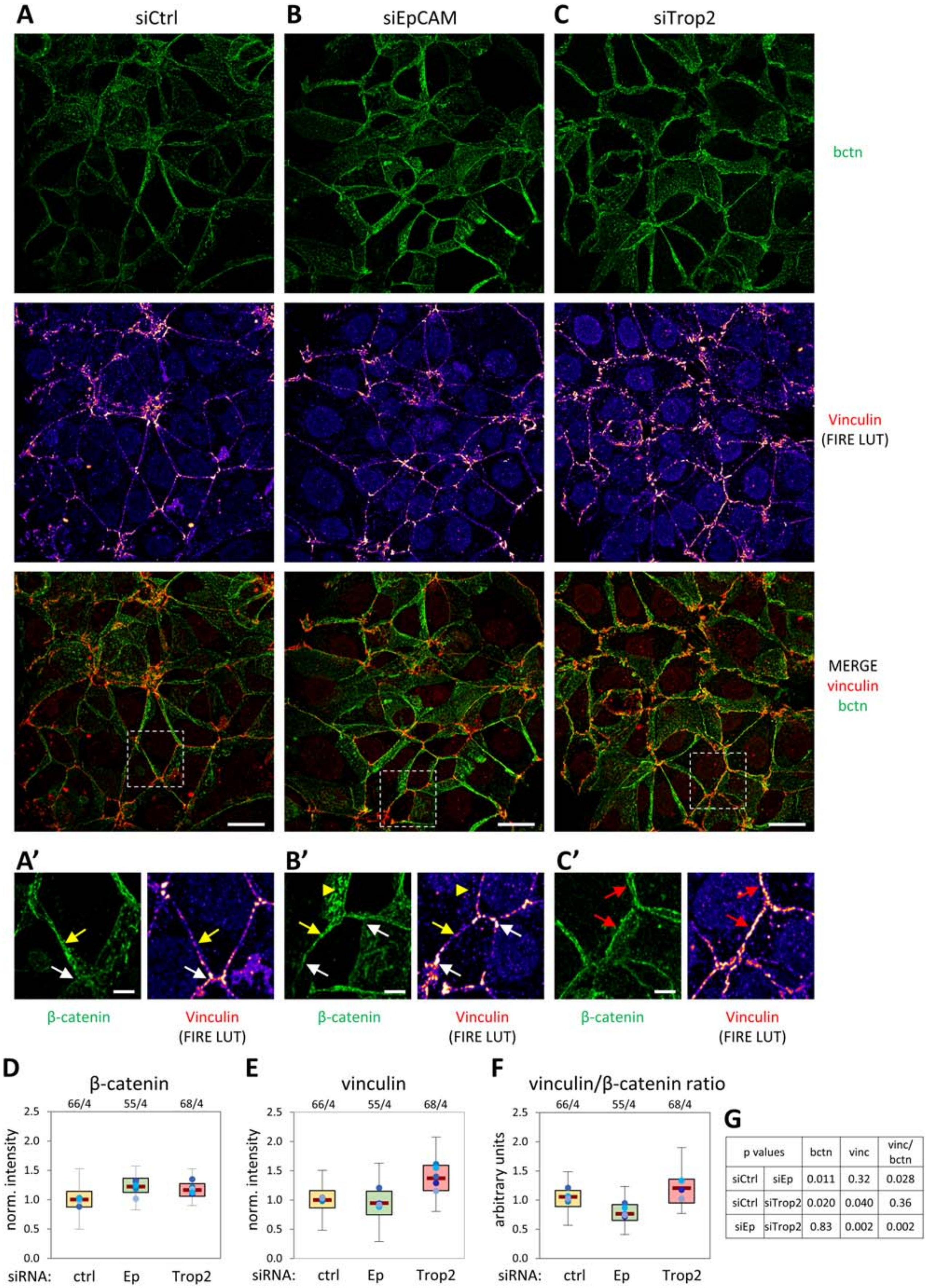

p values		bctn	vinc	vinc/ bctn
siCtrl	siEp	0.011	0.32	0.028
siCtrl	siTrop2	0.020	0.040	0.36
siEp	siTrop2	0.83	0.002	0.002

Figure EV3. Comparison of β-catenin (bctn) and vinculin at cell-cell contacts.

MCF7 cells were laid on a thin layer of collagen. (**A–C**). Maximal projections from confocal z-stacks, excluding the ventral planes containing the FAs. (**A′–C′**). Enlarged fields from A-C. White arrows, membrane regions with high vinculin and low bctn. Yellow arrows, membrane regions with low vinculin and high bctn. Red arrows in (**C′**), membrane regions with both high vinculin and high bctn. Scale bars: (**A–C**), 20 µm; (**A′–C′**), 5 µm. (**D–G**). Quantification of (**D**) bctn, (**E**) vinculin, and (**F**) vinculin/bctn ratio. The box plots show the interquartile range (box limits), median (center line), and min and max values without outliers (whiskers). Numbers of fields/biological replicates indicated above graphs. One-way non-parametric ANOVA (Kruskal–Wallis Test) on experiment averages, followed by Dunn post hoc test. *p* values are given in (**G**).

© The Author(s)

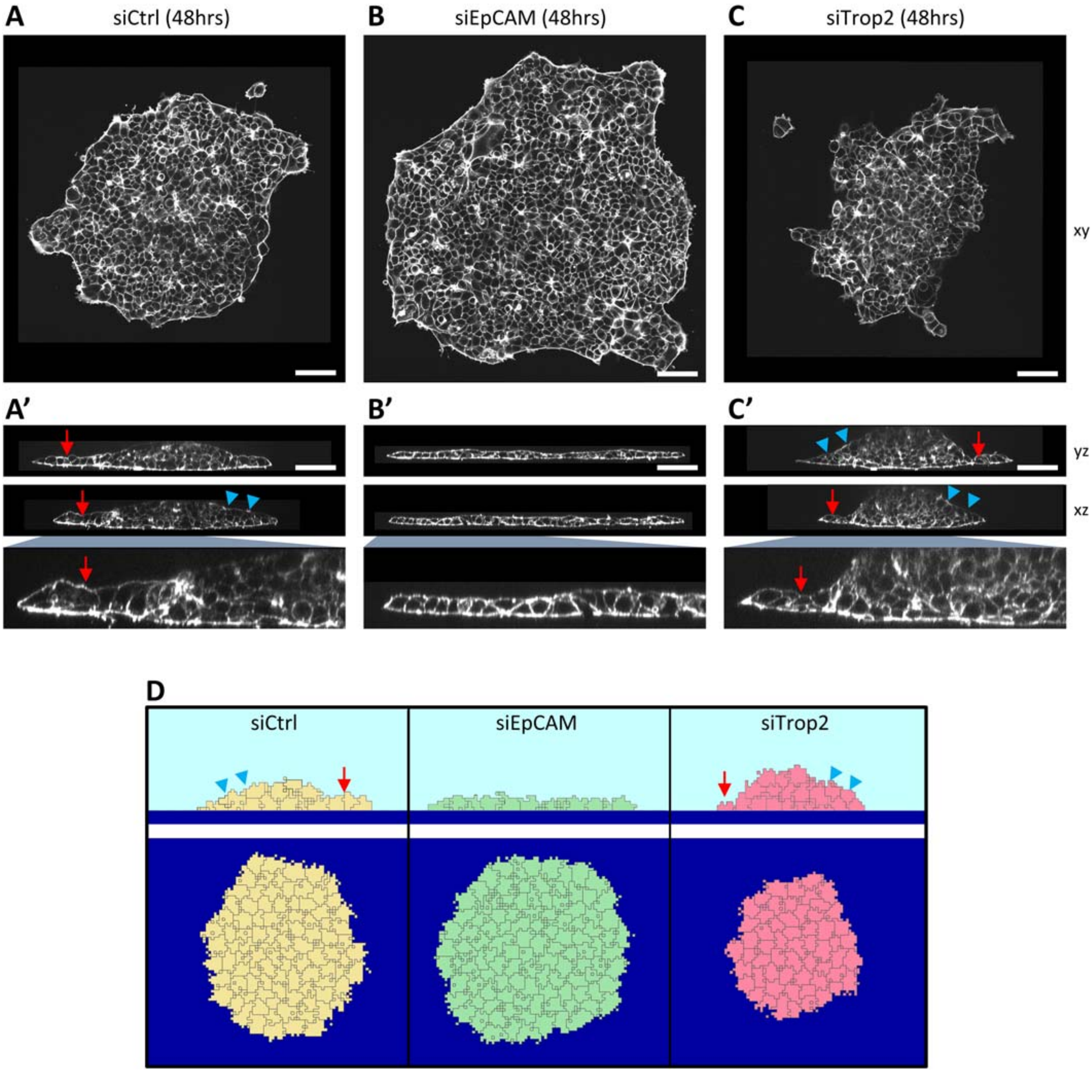

Figure EV4. Spreading of MCF7 spheroids after 48 h and corresponding simulation.

(**A–C**) Confocal images from spheroids fixed and labeled with phalloidin. Images are representative of 16 spheroids per condition, from four experiments. (**A–C**) Single horizontal planes correspond to the maximal area. (**A′–C′**) Vertical projections (yz and xz) and enlargement of xz. All spheroids have spread further and thinned compared to 24 h (Fig. 1). EpCAM KD spheroids have reached maximal extension, forming a highly coherent monolayer. All tissues have remained largely compact as indicated from the smooth dorsal surface in vertical projections, although in controls and Trop2 KD, portions tend to protrude (red arrows, smooth portions marked by blue arrowheads). Scale bars: 50 μm. (**D**) Projections from CompuCell3D simulation after 600 iterations (i.e., twice longer than in Fig. 5). The actual morphology of the spheroids is very well recapitulated, including the monolayer for EpCAM KD and the irregular profile for control and siTrop2 conditions (compare red arrows and blue arrowheads).

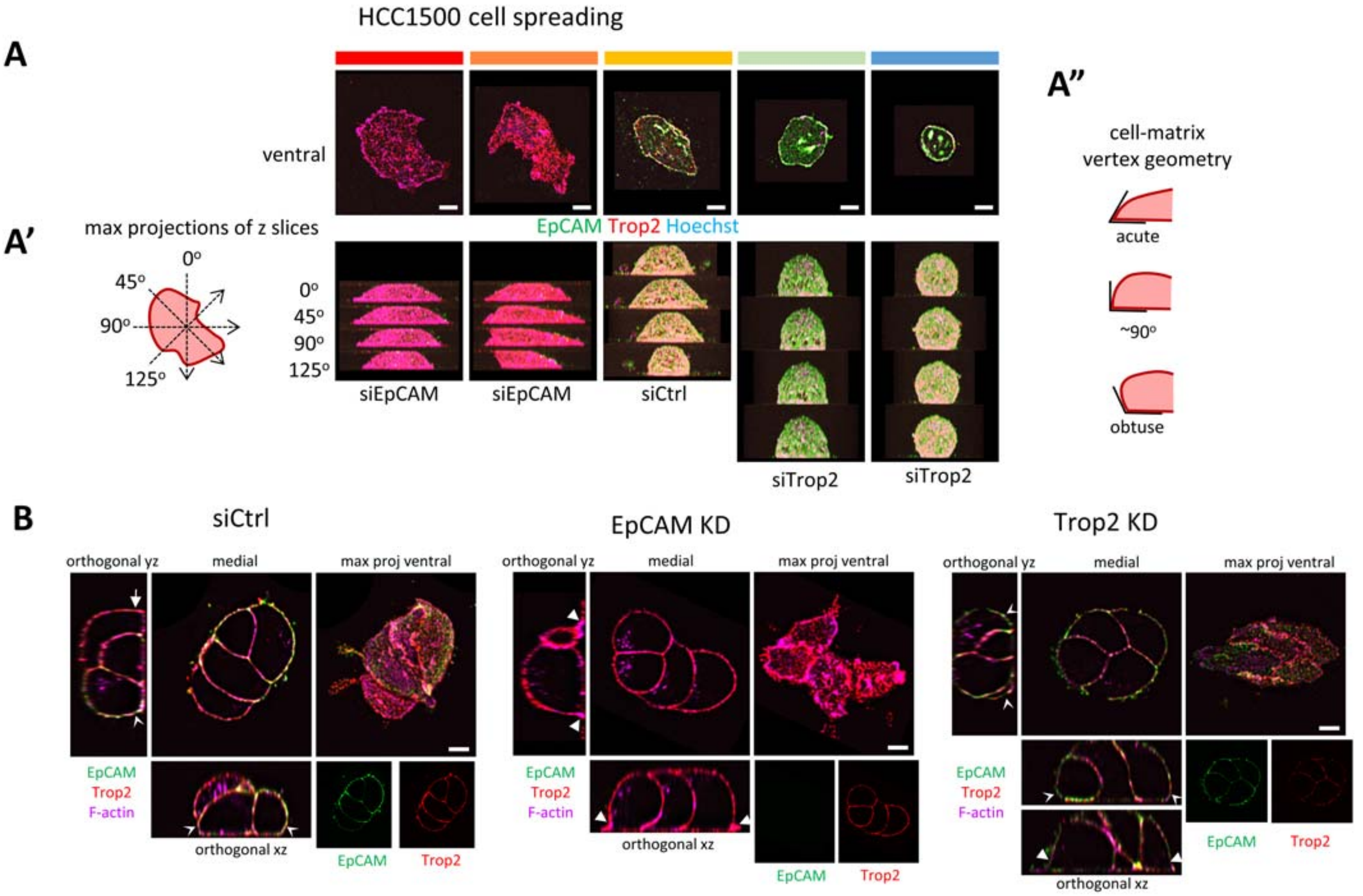

C

Category		Score	Combined edge geometries from four profiles (8 edges)
Spread	Full	6	All acute
	High	4	Most acute
	Moderate	2	All ~90° or weakly acute<br>Half ~90°, half obtuse<br>Part obtuse, part acute<br>Half acute, half ~90°
	Weak	1	Most obtuse, rest ~90° or weakly acute
Round		0	All obtuse

D

P values	SC	Doublets	Groups
siK-siEpCAM	0.0003	0.35	0.006
siK-siTrop2	0.33	0.022	0.93
siEpCAM-siTrop2	7E-06	0.002	0.004

Figure EV5. Analysis of morphology phenotypes of HCC1500 cells.

HCC1500 cells transfected with siCtrl, siEpCAM, or siTrop2, laid on fibrillar collagen, were surface immunolabelled for EpCAM, Trop2 and stained for F-actin (Phalloidin). (**A**) Representative examples of single cells, selected to cover the range of degree of spreading. Scale bars, 5 µm. (**A'**) Orthogonal maximal projections in four orientations. (**A''**) These projections were used to observe the geometry at the contact with the matrix substrate, specifically the angle formed by the free cell edge and the substrate interface. (**B**) Examples of siCtrl, siEpCAM, and siTrop2 groups of cells. Each multiple panel includes merged images of a medial horizontal plane, a maximal projection of bottom (ventral) planes, and two orthogonal views xz and xy. Arrowheads point to the free-edge substrate vertex. Filled arrowhead: acute angle; arrow: right angle; convex arrowhead: obtuse angle. For the Trop2 KD example, two xz slices are shown, one with obtuse acute angles (blue arrowhead), one with acute angles (blue arrow). Scale bars, 5 µm. Separate EpCAM and Trop2 channels of the median plane are shown as small inserts for visualization of their respective levels under control and depletion conditions. (**C**) Morphological classification. Single cells and cell groups were classified into five categories of degree of spreading, from round to fully spread, based on the geometry at the substrate vertex. Because of the highly irregular shapes, multiple combinations were pooled in the intermediate categories. (**D**) Statistical analysis corresponding to the results shown in the main Fig. 7C. *P* values from ANOVA analysis followed by Tukey-HSD post hoc test, obtained by allocating to each category a score (0,1,2,4,6). Results were essentially the same using different scales (e.g., 0, 1, 2, 3, 4).

© The Author(s)
